# Enhancing Alzheimer Disease risk prediction using machine learning for Pathway Polygenic Risk Scores (p‐PRS)

**DOI:** 10.1002/alz.091886

**Published:** 2025-01-03

**Authors:** Farid Rajabli, Vanessa Aguiar‐Pulido, Kyle Scott, Briseida E. Feliciano‐Astacio, Kara L. Hamilton‐Nelson, Larry D. Adams, Pedro R. Mena, Katrina Celis, Jose Javier Sanchez, Patrice L. Whitehead, Michael B. Prough, Heriberto Acosta, Katalina F McInerney, Jeffery M. Vance, Michael L. Cuccaro, Gary W Beecham, Margaret A Pericak‐Vance

**Affiliations:** ^1^ John P. Hussman Institute for Human Genomics, Miller School of Medicine, Miami, FL USA; ^2^ Department of Computer Science, University of Miami, Miami, FL, USA, Miami, FL USA; ^3^ John P. Hussman Institute for Human Genomics, University of Miami Miller School of Medicine, Miami, FL USA; ^4^ Universidad Central del Caribe, Bayamón, PR USA; ^5^ John P. Hussman Institute for Human Genomics, Miami, FL USA; ^6^ John P. Hussman Institute for Human Genomics, University of Miami Miller School of Medicine, Miami, FL, USA, Miami, FL USA; ^7^ Carribean Center for the study of memory and cognition, San Juan, PR USA; ^8^ University of Miami Miller School of Medicine, Miami, FL USA; ^9^ 1501 NW 10th Avenue, Miami, FL USA

## Abstract

**Background:**

Polygenic Risk Scores (PRS) are important in predicting disease risk and are usually rely on markers selected by thresholding p‐values from genome‐wide association studies (GWAS). In traditional approaches, one single model is built to calculate risk scores, employing effect size to determine additive risk. However, this traditional method overlooks potential interactions between genetic loci resulting in reduced prediction power. To overcome these limitations, we propose an interpretable machine learning approach based on pathway‐level PRS (p‐PRS). Our approach improves prediction accuracy of PRS and generates both pathway specific and overall PRS considering possible nonlinear interactions. This advancement opens new avenues in personalized medicine, enabling more accurate disease prediction and prevention strategies.

**Method:**

We used whole genome sequencing data from 652 individuals from the Puerto Rican Alzheimer’s Disease Initiative (PRADI). First, we selected Alzheimer disease (AD)‐related pathways and genes based on those reported in the largest non‐Hispanic White AD GWAS to date (Bellinguez et al. 2021). Then, we applied the Clumping and Thresholding (C+T) PRS approach using Bellinguez et al. summary statistics within each pathway (±20Kb for each gene). Finally, we used random forest with pathway‐level PRS values as input to classify AD vs. control. We assessed the performance of the p‐PRS model based on area under the receiver operating characteristic curve (AUC) using a 70%/30% split for training/testing. The outcomes were compared to a traditional C+T PRS model (AD∼PRS+*APOE*).

**Result:**

p‐PRS improves the performance of the traditional PRS model by 4.2% (p = 5.54E‐32). Performance is centered around AUC = 0.689(SE = 0.0022) for p‐PRS and 0.647(SE = 0.0024) for traditional PRS. PRS by pathway enables determining individual‐level important pathways for accurate classification. Top pathways obtained are related to processes implicated in cognitive deficit, neuroinflammation, neurodegeneration and vesicle mediation.

**Conclusion:**

Utilizing the innovative p‐PRS approach has improved the estimation of AD risk in the PR cohort. The application of interpretable machine learning approaches allows identifying the most relevant pathways for effective risk prediction and classification. Importantly, improved precision will provide more effective actionable risk mitigation strategies, optimize the selection process for clinical trials, and contribute to the development of more personalized treatment interventions.

